# Intellectual disabilities teaching for medical students: a scoping review

**DOI:** 10.1186/s12909-023-04766-4

**Published:** 2023-11-01

**Authors:** Georgia Towson, Stephanie Daley, Sube Banerjee

**Affiliations:** 1 Brighton and Sussex Medical School, Centre for Dementia Studies, University of Sussex, Trafford Building, Room 101, Falmer, BN1 9RY UK; 2 Faculty of Medicine and Health Sciences, University of Nottinham, Nottingham, UK

**Keywords:** Learning disabilities, Intellectual disabilities, Education, Medical students

## Abstract

**Background:**

People with intellectual disabilities are a marginalized group whose health experiences and outcomes are poor. Lack of skill and knowledge in the healthcare workforce is a contributing factor. In England, there is a new legislative requirement for mandatory intellectual disability training to be given to the existing healthcare workforce, including doctors. There is a lack of evidence about effective models of educational delivery of such training in medical schools. We undertook a scoping review to assess the range of intellectual disabilities educational interventions and their effectiveness.

**Methods:**

We included any study from 1980 onwards which reported an educational intervention on intellectual disability, or intellectual disability and autism, for medical students from any year group. Databases searched included PUBMED, ERIC, Scopus and Web of Science as well as searches of grey literature and hand searching two journals (Medical Education and Journal of Learning Disabilities). 2,020 records were extracted, with 1,992 excluded from initial screening, and a further 12 excluded from full-text review, leaving 16 studies for inclusion. Data was extracted, quality assessed, and findings collated using narrative analysis.

**Results:**

We found a variety of intervention types: classroom-based teaching, simulation, placement, home visits, and panel discussions. There was substantial variation in content. Most studies involved lived experience input. Across studies, interventions had different learning outcomes which made it difficult to assess effectiveness. Overall study quality was poor, with high use of non-validated measures, making further assessment of effectiveness problematic.

**Conclusions:**

There is a need for more consistency in intervention design, and higher quality evaluation of teaching in this area. Our review has drawn attention to the variety in teaching on this topic area and further research should focus on updating this review as curriculum changes are implemented over time.

## Background

Healthcare is changing. We have an increasing need to develop and manage the long-term conditions of the 21st Century rather than simply focus on the acute care agenda of the 20th Century. This means the need to develop and deliver care for marginalised and left-behind groups such as those with intellectual disabilities. We know that for this group, life expectancy is significantly lower than for the general population [1]. The lack of routine intellectual disabilities training for the healthcare workforce [2, 3] has been recognised as being a contributing factor to episodes of poor-quality care where health outcomes for people with intellectual disabilities are severely compromised [4]. Approximately half of all deaths of people with intellectual disabilities have been deemed to be avoidable, compared to less than a quarter of those in the general population [1].

In response to these concerns, a legislative requirement for health and social care staff to undertake mandatory intellectual disability and autism training has been embedded in statue in England [5]. It is recognised that doctors along with other healthcare professionals, need to develop the necessary knowledge, attitudes, professional values and skills to deliver good quality and equitable care to people with intellectual disabilities across a range of care settings. There is a lack of consensus about how best to meet this challenge; for doctors in practice, there is a lack of robust evaluation of existing programmes [6]. For doctors in training, where attitudes are potentially more malleable and open to change, the evidence is sparse. One Australian study sought to identify how intellectual disabilities were taught within medical schools [7], and found that lectures were the most common teaching format (67%), with some degree of involvement of people with intellectual disabilities in designing or teaching in about half of all medical schools.

In order to enable the future medical workforce to better meet the needs of people with intellectual disabilities there is a need to ensure that effective educational interventions are used. To support this, we carried out a scoping review to assess the range of intellectual disabilities educational interventions used in medical education and their effectiveness.

## Methods

A scoping review was chosen in order to categorise existing, predictably disparate, literature, and identify knowledge gaps [8]. A protocol was developed following PRISMA-ScR guidance [9].

The objective of this scoping review was to understand the extent and type of evidence on intellectual disabilities teaching for undergraduate medical students.

The specific review questions were:

1. What educational interventions are being delivered in intellectual disabilities for medical students?
2. How effective are these educational interventions at improving student learning outcomes such as knowledge and confidence?

### Eligibility criteria

The inclusion criteria for this scoping review included studies reporting an educational intervention on intellectual disability, or intellectual disability and autism. It included medical students across any year group at any medical school worldwide. Studies were only included if written in English and were published since 1980, as this is when the diagnosis of autism spectrum disorder was formally recognised, and The National Joint Committee on Learning Disabilities defined a clear criteria for ‘learning disabilities’.

Studies were excluded if they covered the qualified workforce, junior doctors, or other healthcare students but not medical students Studies were also excluded if they reported mixed groups including medical students but did not differentiate between student type in results. Studies looking at autism teaching only, or studies reporting on both physical and intellectual disability where results for both areas were undifferentiated were also excluded, as well as studies with no post intervention outcome evaluation of any design type.

### Search strategy

An initial limited search of Medline and ERIC was undertaken to identify papers on the topic. The text words contained in the titles and abstracts of relevant articles, and the index terms used to describe the papers were used to develop a full search strategy for Medline. A final search strategy was developed with guidance from a specialist librarian and is shown in Table 1.

**Table 1:** Search terms and results

#	Search term	Results
1	Learning Disabilities/	14,438
2	(disabil* or “learning disab*” or “learning impair*” or “learning deficit” or “developm* disab*” or “special needs”).mp. [mp = title, abstract, original title, name of substance word, subject heading word, floating sub-heading word, keyword heading word, organism supplementary concept word, protocol supplementary concept word, rare disease supplementary concept word, unique identifier, synonyms]	313,177
3	1 or 2	313,177
4	curriculum/ or education, medical/ or education, medical, undergraduate/ or teaching/	178,810
5	(“medic* education” or teach* or curricu* or train* or learn*).mp. [mp = title, abstract, original title, name of substance word, subject heading word, floating sub-heading word, keyword heading word, organism supplementary concept word, protocol supplementary concept word, rare disease supplementary concept word, unique identifier, synonyms]	1,328,619
6	4 or 5	1,355,871
7	Students, Medical/	38,690
8	(“medic* students” or “*undergrad* medic* student*” or “undergrad* student*” or “prelicense student*”).mp. [mp = title, abstract, original title, name of substance word, subject heading word, floating sub-heading word, keyword heading word, organism supplementary concept word, protocol supplementary concept word, rare disease supplementary concept word, unique identifier, synonyms]	51,934
9	7 or 8	68,535
10	3 and 6 and 9	385
11	limit 10 to (english language and yr="1980 -Current”)	356

Databases searched included PUBMED, ERIC, Scopus and Web of Science. Additionally, sources of unpublished studies/grey literature were hand searched for in Google Scholar, Open Grey as well as two journals BMC Medical Education and Journal of Learning Disabilities; which were deemed most relevant to our topic. The reference list of all included sources of evidence were screened for additional studies. Authors of papers were contacted when full text was not available online or from the library. The search was conducted on the 24th of November 2021. *Study selection*.

Following the search, all identified citations were collated and uploaded into Endnote Reference software (version 20) and duplicates were removed. Following a pilot test; of where both reviewers (GT and SD) had 100% agreement on 10% of the identified citations, titles and abstracts were screened by one reviewer (GT) for assessment against the inclusion criteria for the review in Rayyan CQRI software [10]. Potentially relevant sources were retrieved in full, and their citation details also imported into Rayyan.

### Data extraction

**Fig. 1:**
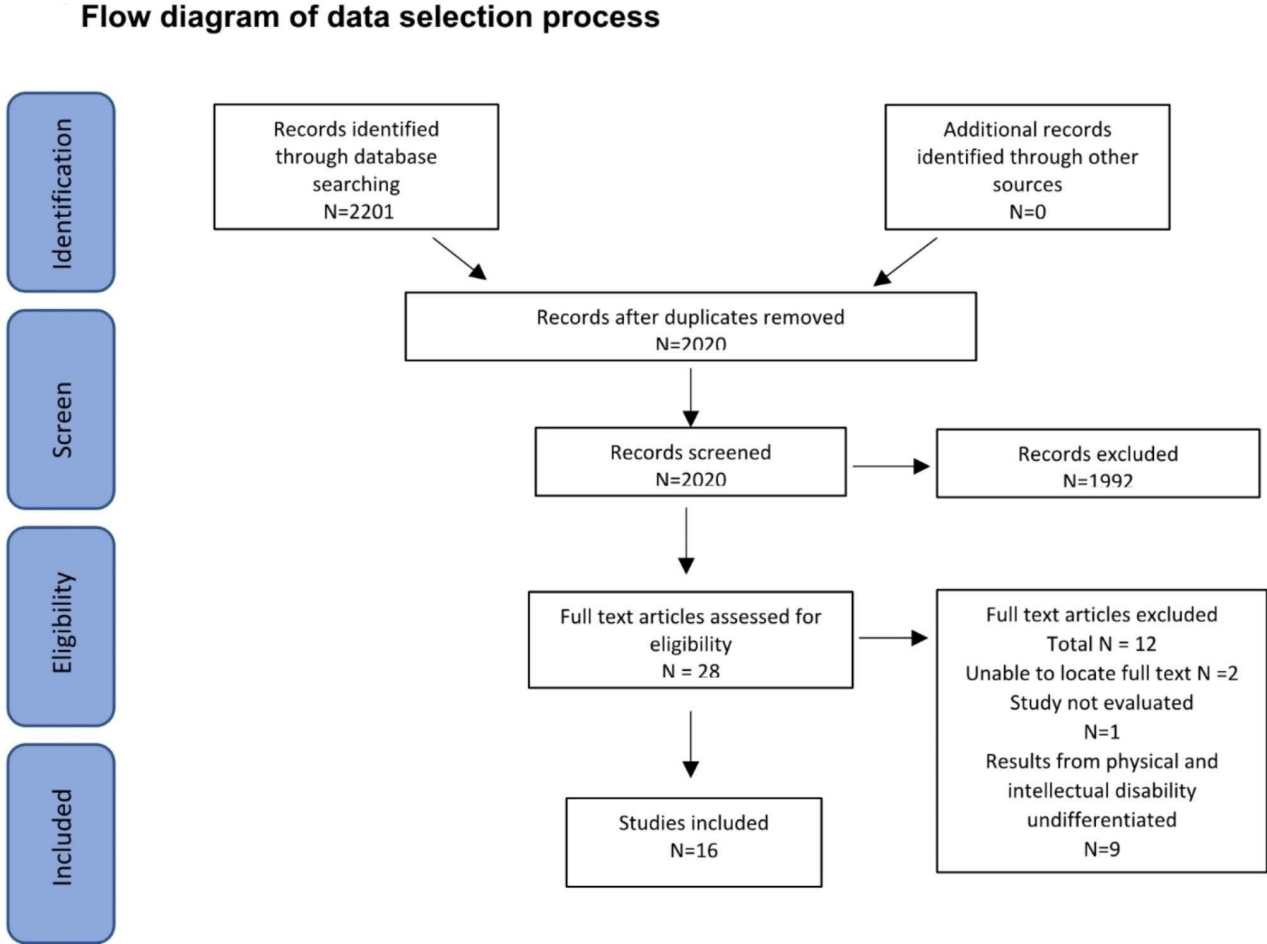
Flow diagram of data selection process

The full text of selected citations were assessed in detail against the inclusion criteria by one reviewer (GT) with a second researcher (SD) reviewing 15% of the references. 2,020 studies were found in the original search after duplicates had been removed. 28 of these were suitable for full text review, where 12 were then excluded, leaving 16 papers. Reasons for exclusion of sources of evidence at full text that did not meet the inclusion criteria were recorded and can be seen in Fig. 1.

Any disagreements between the reviewers at each stage of the selection process were resolved through discussion. Charted data was entered into a Microsoft Excel spreadsheet. The data extraction tool was modified and revised during the data extraction process; once to allow for a more detailed description of the critical appraisal tool, and on a further occasion to allow for a more detailed narrative analysis of outcomes to be collated.

### Data items

Study demographics such as author, title of paper, year of study, design and country of origin, and aim of study were extracted, as well study participant year, sample size, and critical appraisal scores. Intervention duration, whether there was lived experience, whether the intervention was compulsory, intervention focus, and intervention category were also extracted. Studies with similar educational interventions were grouped together to form the intervention category. This was agreed by both researchers. Qualitative themes, how the data was analyzed, data collection methods and sample size made up the qualitative extraction. Quantitative data included measures used and intended measurement e.g. attitude/knowledge/comfort, as well as statistical significance, mean and standard deviation. Where papers only provided descriptive measures, percentages and ratios were reported.

### Critical appraisal

Two critical appraisal tools were used to assess all studies, a modified Kirkpatrick scale [11] and Best Evidence Medical Education (BEME) [12]. The modified Kirkpatrick scale developed by Adirim and colleagues [6] involved grading papers between 0 and 4, with zero indicating student satisfaction assessment only, and four evidencing direct impact on patient outcomes or organisational structure (6). This was followed by the BEME appraisal, which was split into two Sect. [13], first assessing the strength of findings and second, the overall importance of the study [14]. Papers were given two grades which ranged from 1 to 5, with 1 indicating ‘no clear conclusions’, to 5 indicating ‘unequivocal results’ [13]. Overall importance of the paper was also assessed on a scale from 1 to 5, with 1 being ‘papers with numerous deficiencies in the rigor or appropriateness of the methodology or the statistical analysis’, and 5 being ‘papers with generalisable findings, rigorous methodology, adequate sample size and appropriate statistical analysis’ [14]. One reviewer (GT) appraised all papers using these two tools, and a second researcher (SD) appraised 15% (n = 3) of all papers, where there was 100% agreement. Papers were not excluded due to a low BEME score, but scores on the quality of papers are reported.

### Synthesis of results

Education interventions were grouped into categories based on similarity of the intervention being described [15]. Studies were also grouped by specific outcome measured, to see if certain intervention types improved a specific outcome more than another.

## Results

### Study demographics

Sixteen papers met the inclusion criteria and were included in the review. Country of origin included the UK (n = 7) [16–22], the USA (n = 5) [23–27], Canada (n = 2) [28, 29], and Australia (n = 2) [30, 31]. Five studies were conducted in the 1990s [17, 22, 24, 27, 31], one in the 2000s [30], nine in the 2010s [18–21, 23, 25, 26, 28, 29] and one in the 2020s [16]. Most papers were conducted solely with medical students, however one included physical therapy students [23], one included physical therapy, occupational therapy, and nursing students [29], and one also included those already in the workforce; nursing assistants, nurses, and resident physicians [24].

Seven studies measured outcomes using mixed-methods (16, 22, 26 − 8, 30, 31) eight quantitatively [17–21, 23, 24, 29] and one qualitatively [25]. Only one study [28] had a control group and utilised a randomised control trial design.

Eleven of the interventions involved direct lived experience in the delivery of teaching [16–19, 22, 23, 25, 27, 28, 30, 31]. Two involved videos of people with intellectual disability [20, 29], one involved parents of a person with a disability [26], and two studies did not report lived experience inclusion [21, 24]. Ten of the interventions were elective [18, 19, 22–24, 26, 28–31], with five compulsory [16, 20, 21, 25, 27], and one unclear [17]. Table 2 provides an overview of the 16 studies included in this review, giving key characteristics.

**Table 2:** Summary of study characteristics

Author	Year of Study	Design	Origin	Study population	Sample size	Disability focus	Intervention Category	Lived experience (Y/N)	Compulsory (Y/N)
Abdi & Metcalf	2020	Mixed methods	Wales	Year 4	66	Intellectual	Communication skills workshop	Y	Y
Coret et al.	2018	Mixed methods - control	Canada	Year 1	27	Intellectual and Developmental	Simulation with SP’s	Y	N
Garavatti et al.	2018	Quantitative	America	Year 2	20*	Developmental (including intellectual)	Simulation with SP’s	Y	N
Hall & Hollins	1996	Quantitative	England	Unknown	28	Down syndrome	Drama workshop	Y	Unknown
Harper & Wandsworth	1992	Quantitative	America	Year 2	12*	‘Mental retardation’	Classroom based	Unclear	N
Harwood & Hassiotis	2014	Quantitative	England	Year 4	69	Intellectual	Classroom based	N**	Y
Jones et al.	2015	Quantitative	Canada	Year 2	94*	Intellectual	Classroom based	N**	N
Jones & Donald	2007	Mixed methods	Australia	Year 4	26	Intellectual	Placement	Y	N
Karl et al.	2013	Qualitative	America	Year 3	144	Developmental	Placement	Y	Y
May	1991	Mixed methods	Scotland	Year 2	24	‘Mental retardation’	Placement	Y	N
Sheppard et al.	2017	Mixed methods	America	Year 2	112	Mixed- Autism/Down Syndrome/Intellectual Disability	Panel discussion	N**	N
Sinai et al.	2013	Quantitative	England	Year 4	136	Intellectual	Classroom based	N	Y
Thomas et al.	2014	Quantitative	England	Year 4	47	Intellectual	Simulation with SP’s	Y	N
Tracy & Graves	1996	Mixed methods	Australia	Year 1	25	Developmental	Placement	Y	N
Watkins & Colgate	2016	Quantitative	Wales	Year 3	23	Intellectual	Simulation with SP’s	Y	N
Widrick et al.	1991	Mixed methods	America	Year 3	39	‘Mental retardation’	At home visits	Y	Y

*Footnote:

*Garavatti et al. also included 20 physical therapy students in their study. Harper and Wandsworth also included 12 nursing assistants, 9 nurses and 11 resident physicians

Jones et al. 2015 also included psychology, occupational therapists, psychical therapists and nursing students

**Harwood and Hassiotis and Jones et al. 2015 had lived experience in videos but not in person delivery. Sheppard et al. involved parents of those with a disability

### Intervention categories

Studies were categorized based on their main intervention type, according to the most commonly used terms within the paper. Four studies [18, 19, 23, 28] were categorized as a simulation with a standardized patient. This included a workshop style structure where students would visit ‘stations’ and perform a clinical encounter role play with someone with an intellectual or developmental disability, followed by a debrief or reflective session. A further four studies [20, 21, 24, 29] were classified as classroom based, which included didactic lectures, e-learning, seminars, self-study instructional text, and videos. Another four studies [22, 25, 30, 31] were placement-based, which included spending a varied amount of time (one day to eight weeks) in a variety of environments such as a school for those with an intellectual disability, primary care or hospital services for people with intellectual disabilities. One study [17] was a drama workshop with a theatre company of adults with Down’s Syndrome, and one study [16] was a communication workshop with a session by the speech and language therapy team, involving in person case studies with a simulated patient. Another category was a Question and Answer panel discussion [26], with parents of children with a disability. The final category was home visits [27] where students would visit a child with a disability and their parents in their own home as a one-off meeting, in between a preparatory and reflective lecture. Students were not advised of the specific disability of the child before visiting them, in order to avoid pre-assumptions.

### Outcomes measured

A variety of outcomes were measured; both quantitatively and quantitatively, across all studies. These included satisfaction, knowledge, attitudes, comfort, communication, skill/practice, understanding, professional identity, and awareness. Table 3 describes the outcomes measured and the key findings from each study, which are summarized in the following section.

**Table 3:** Outcome measures and key findings of each study

Author, Year	Target outcome	Research tool	Key Findings/discussion
Abdi & Metcalf, 2020	Attitudes	ADTP-B and semi structured interviews	Paired sample t tests showed scores were significantly higher in the ATDP-B questionnaire after the teaching session (M = 122, SD = 17.2 to M = 115, SD = 14.5) t (65) = 6.20, p < 0.001. The average difference in ATDP-B scores before and after the teaching session was 6.92. Qualitative results helped to expand upon the quantitative, showing that students felt the teaching session allowed them to develop professional identity, and overcome communication barriers.
Coret et al., 2018	Communication and comfort	Focus groups, own survey	Both control and narrative groups improved in comfort, confidence and competence but mean rating scores were higher for students in the narrative group. However, these trends are descriptive only. Qualitative findings highlighted the need for adaptable communication styles and the universality of person-centred communication.
Garavatti et al., 2018	Attitude and comfort	RSA, ADTP, IDP	The ATDP results showed improvement pre and post (84 vs. 81.6) p < 0.05, as well as the IDP (70.9 vs. 65,6) p = 0.01, however these were not significant. Medical students improved their comfort levels, with the RSI improving from 83.45 to 73 (p < 0.001).
Hall & Hollins, 1996	Attitude	Own survey	For all statements, there was more agreement with positive statements and less agreement with negative statements after the workshop than before it. Seven of the changes achieved statistical significance. For example 1. People with DS are poor communicators median 23 v 14 pre post (P < 0.001). 7. People with DS tend to be frightening median 8 v 6 pre post (p = 0.05).
Harper & Wandsworth, 1992	Knowledge and skill	Own survey	Change in medical students’ knowledge did not show significance. Medical students did significantly improve on elements of communication skill after taking part in the intervention and at 6 week follow up e.g. medical students increased their use of open-ended questions (from 32.3 to 50.1 mean seconds, (t-test, P < 0.05), whilst decreasing the amount of time they spent using declarative sentences 143.4 to 105.4 mean seconds (t-test, P < 0.05).
Harwood & Hassiotis, 2014	Satisfaction and knowledge	Own survey	This study focused on satisfaction and knowledge, but did not measure significance. The study indicated that students showed a positive outlook towards those with an intellectual disability and appreciated the need for training (91%).
Jones et al., 2015	Knowledge, attitudes, skill	Own survey	Attitudes towards those with an intellectual disability did not show statistically significant change and stayed neutral. Skill change was observed (pre post 68% vs. 73%) but this also showed no statistical significance. Medical students’ knowledge scores did show a significant difference between pre course (M = 0.50, SD = 0.23) and post course (M = 0.69, SD = 0.23); t(93) = − 7.407,p ≤ 0.01.
Jones & Donald, 2007	Satisfaction and understanding/importance	Open questionnaire, own survey	This study had a large focus around students experience of the intervention rather than its effect. Study did not measure significance but suggested improvement in understanding, with positive satisfaction. For example, all 21 participants strongly agreed that the placement gave them a better understanding of children with special needs.
Karl et al., 2013	Attitudes, comfort, communication	Reflective questionnaire	Qualitative analysis showed that students had overcome communication barriers, improved their attitudes and comfort level whilst working with people with disabilities and thought more about the organizational structure in the medical environment.
May, 1991	Satisfaction and attitudes	Questionnaire/word association	There was no significant change on attitude after the intervention. Word association showed that participants chose negative or positive words to represent someone with an intellectual disability in the same ratio as the beginning. Students’ choice of ‘positive words’ were happy friendly loving and affectionate which can constitute to the negative stereotyping of those with a disability. The number of students wanting to work with someone with an intellectual disability decreased from 4 (17%) to 2 (12%).
Sheppard et al., 2017	Knowledge and understanding/importance	Discussion essays, own survey	The differences between the students pre and post assessment responses for the Level 1 questions (basics) showed significant increases in correct responses. For level 2 questions (application), the changes between the pre and post responses were statistically significant for correct responses, incorrect responses and do not know responses. Many students changed their answer choice from do not know to either the correct or incorrect answer after the program. Qualitative results helped to give more understanding of the physician, and helped students understand the importance of learning this topic.
Sinai et al., 2013	Knowledge and attitudes	CLAS-MR, own survey	There was no significant change in any of the attitude subscales between the beginning (T1) and the end (T2). Significant improvements were found when comparing answers to some of the knowledge based questionnaires from the beginning to the end. These included correctly identifying dyslexia is not a learning (intellectual) disability (T1 = 98 (73%) T2 = 54 (44%) P value = 0.001 and recognising the definition of learning (intellectual) disability (T1 = 105 (80%) Tw = 121 (95%) P value = 0.001. Analysis of paired data showed similar results apart from no significant difference being found in understanding the definition of learning disability.
Thomas et al., 2014	Comfort, communication, skill	Own survey based on ‘healthcare provider questionnaire’	There was significant improvement in the scores in communicating with people with no disability (< 0.005), people with mild intellectual disability (p < 0.001) and people with severe intellectual disability (< 0.001). The mean scores were significantly higher for the severe disability scenario than the mild disability scenario, indicating the impact of the training was higher of terms of managing patients in this group. There were also significant improvements in students perceived skill when treating people in all three groups; no disability, mild disability or severe disability (1.43 (95% CI 0.50–2.35; t(46) = 3.14, P = 0.002), 6.47 (95% CI 5.27–7.67; t(46) = 10.82, P < 0.001) and 8.87 (95% CI 7.49–10.2; t(46) = 12.96, P < 0.001) respectively). There was a significant improvement in the type of clinical approach adopted by students in managing patients with none, mild or severe disability post-training. The corresponding mean differences were: 1.19 (95% CI 0.42–1.96, t(46) = 3.12, P < 0.005), 3.77 (95% CI 2.69–4.84, t(46) = 7.05, P < 0.001) and 5.48 (95% CI 4.12–6.84, t(46) = 8.13, P < 0.001) respectively.
Tracy & Graves, 1996	Satisfaction and attitudes	Own survey, questionnaire/word association	Study did not measure significance but indicated students thought the intervention fulfilled their expectations (86% of 25) and changed feelings and beliefs (92% of 25). Students discussed the intervention contributing to their personal identity, as well as having more insight into the topic of learning disability. Word association identified a change from negative to positive outlooks ranging from 8–60% when using words to describe children with disabilities and 15–50% when using words to describe adults with disabilities.
Watkins & Colgate, 2016	Knowledge and understanding	Own survey	Students mean score within the knowledge domain significantly improved pre post (14.87 vs. 10.65, p < 0.001). Students mean score within the affect and understanding domain also significantly improved (13.7 vs. 10.52, p < 0.001).
Widrick et al., 1991	Attitude and awareness/insight	PMRS, student log book review	Students improved their attitudes pre to post test (35 vs. 41, p < 0.001). Students also increased their expectations of capabilities of all three subgroups; those with a mild, moderate or severe intellectual disability. Qualitative results indicated students had improved their awareness and insight into intellectual disability.

### Satisfaction

Four studies [20, 22, 30, 31] measured how satisfied the students were with the intervention, all using non-standardized measures they had developed. None of these studies measured statistical significance of this outcome, and instead presented either percentages and agree/disagree statements [20, 30, 31], or mean scores across a five point scale [22].

### Knowledge

Six studies [19–21, 24, 26, 29] measured whether the students had increased their knowledge after taking part in the intervention. All studies used non validated measures, and all developed their own measurement tool. Five of these studies assessed knowledge using pre-post measures [19, 21, 24, 26, 29], with one [20] measuring knowledge post intervention only.

### Attitudes

Nine studies [16, 17, 21–23, 25, 27, 29, 31] measured attitude change, seven quantitatively [16, 17, 21–23, 27, 29], one qualitatively [25], and one using mixed methods [31]. The following standardised measures were used:

1. Attitudes Towards Disabled People-B (ATDP-B) [29, 16, 23];
2. The ‘Interactions with Disabled People’ scale (IDP) [23, 32];
3. Prognostication about Mental Retardation scale (PMRS) [27, 33];
4. Community Living Attitudes Scale- Mental Retardation (CLAS-MR) [21, 34].

Four studies [17, 22, 29, 31] used non-standardised measures, including a word association method [22]. The qualitative study measured attitude change through reflective questions [25].

### Comfort

Change in students’ comfort of being able to treat someone with an intellectual or developmental disability was measured in four studies: two quantitatively [23, 28], one qualitatively [25], and one through mixed methods [18]. One study measured comfort using the Rehabilitations Situations Inventory (RSA) [23, 35]. Two studies used their own non-validated survey [18, 28] and another measured comfort through reflective questions [25].

### Communication

Improvement in communication skills was measured in four studies. Two used qualitative methods [16, 25], one mixed- methods [28], and one quantitative methods [18]. Two used their own non-validated measure [18], with one using focus groups [28]. One used reflective questionnaires [25], with the final using a combination of semi structured interviews and focus groups [16].

### Skill/practice and understanding/importance

An improvement in skill (assessed through educator observations and self-reports) and understanding the importance of learning about intellectual disability were each measured separately in 3 studies. All three studies [18, 24, 29] used their own non-validated survey to measure an improvement of skills. Two of the studies [19, 30] also looked at student understanding of the importance of learning about intellectual disabilities measures through a non-validated survey, and one study analysed discussion essays qualitatively [26].

### Intervention effectiveness

All intervention types showed a positive trend when measuring satisfaction, however due to the use of non-validated measures, no studies reported statistical significance. Of the six studies measuring knowledge, four showed statistical significance [19, 26, 29, 31]. These fell into the categories of classroom based (n = 2) panel discussion (n = 1), and simulation with simulated patients (n = 1).

Of the nine studies measuring attitude change, only three studies showed a statistically significant change pre-post intervention [16, 17, 27]. The three intervention types were a communication workshop; drama workshop, and home visits. Two placement-based interventions reported attitude change through qualitative questionnaires or word association [25, 31]. None of the classroom-based interventions demonstrated attitude change.

Three studies [18, 23, 25] showed that medical students comfort levels working with people with intellectual disabilities improved post-intervention, through either significant quantitative results or qualitative thematic analysis. These fell into the categories of simulations with a simulated patient (n = 2), and placement-based (n = 1). When looking at improved communication, statistical significance was found in one set of quantitative data [18] for a simulation. All the studies who measured this qualitatively (drama workshop, simulation, and placement-based) identified positive change [16, 25, 28]. Only one of these studies also measured outcomes quantitatively however outcomes did not reach statistical significance [28]. Two studies [18, 24], a simulation and classroom-based activity, showed statistical significance in changing and improving medical students’ skill and their implementation in practice. This was assessed through educator observation and self-reports. One study [29], also classroom-based, was not statistically significant in improving skill.

When looking at understanding the importance of learning about intellectual disability, one study showed statistical significance in a simulation [19], whilst another showed a positive trend through a placement but did not measure statistical significance [30]. Understanding was also shown as a qualitative theme in one study [26], which was a panel discussion with parents of people with a disability.

### Qualitative outcomes

Two themes arose from qualitative studies; both found in two different studies; which were ‘professional identity’ and ‘awareness and insight’. Both Abdi (2020) and Tracy (1996) found that their intervention (communication skills workshop and a placement) had allowed students to develop a more professional identity as they were taught how to overcome communication barriers [16] and therefore identify more as a doctor, as well as perception of personal development and enhanced professional identity [31]. One study [31] reported that placement-based interventions facilitated awareness of the family life of someone with an intellectual disability. Another study [27] similarly said that home visit-based interventions allowed students to grow their understanding of what living with an intellectual disability was like and see intellectual disability in ‘real life’.

### Quality assessment

The overall quality of studies meeting the inclusion criteria was low, with no papers scoring a 5 on either BEME score. 100% of papers scored a 3 or below on importance of findings, with 50% of studies scoring 3, 37.5% scoring 2 and 12.5% scoring 1. 56.25% of studies scored 3 or below on importance of overall paper, with 43.75% of studies scoring 4, 25% scoring 3, 18.75% scoring 2 and 12.5% scoring 1. 87.5% of all studies also scored a 2 or below on the Kirkpatrick score, with one study scoring a 1, and one study scoring 0. Quality scores are shown in Table 4.

**Table 4:** Quality Assessment Scores

Author	BEME score Findings	BEME score Overall importance	Kirkpatrick score
Abdi & Metcalf, 2020	3	4	2
Coret et al., 2018	2	2	2
Garavatti et al., 2018	2	3	2
Hall & Hollins, 1996	3	2	2
Harper & Wandsworth, 1992	2	3	3
Harwood & Hassiotis, 2014	1	1	2
Jones et al., 2015	3	4	2
Jones & Donald, 2007	1	1	0
Karl et al., 2013	3	4	2
May, 1991	2	3	2
Sheppard et al., 2017	3	4	2
Sinai et al., 2013	3	4	2
Thomas et al., 2014	3	4	3
Tracy & Graves, 1996	2	2	1
Watkins & Colgate, 2016	3	4	2
Widrick et al., 1991	2	3	2

The range of outcomes measured and overall study effectiveness are summarised in Table 5.

**Table 5:** Summary of outcome measured and study effectiveness

Author (Year)	Satisfaction	Knowledge	Attitudes	Comfort	Communication	Skill	Understanding	Professional identity	Awareness/ Insight
Abdi & Metcalf (2020)			S		Q			Q	
Hall & Hollins (1996)			S						
Coret et al. (2018)				NS	Q/NS				
Garavatti et al. (2018)			NS	S					
Thomas et al. (2014)				S/Q	S	S			
Watkins & Colgate (2016)		S					S		
Harper & Wandsworth (1992)		NS				S			
Harwood & Hassiotis (2014)	T	T							
Jones et al. (2015)		S	NS			NS			
Sinai et al. (2013)		S	NS						
Jones & Donald (2007)	T						T		
Karl et al. (2013)			Q	Q	Q				
May (1991)	T		NS						
Tracy & Graves (1996)	T		Q/T					Q	Q
Sheppard et al. (2017)		S					Q		
Widrick et al. (1991)			S						Q

Footnote: S = outcome significant, NS = outcome not significant, T = outcome showed positive trend but did not measure significance, Q = outcome shown in qualitative results

## Discussion

This study set out to identify published intellectual disabilities educational interventions delivered to medical students, and their effectiveness. We found that a variety of intervention types existed, from classroom-based activities to home visits, to panel discussions, but their effectiveness varied. A large majority of the studies involved lived experience input, but very few interventions were a compulsory part of the curriculum. Evaluation of these interventions was often conducted with non-validated surveys, and overall study quality was poor, making the assessment of effectiveness difficult.

The most common teaching methods were placements, simulations with simulated patients, or classroom-based activities, but there was a large variety of interventions with no set ‘structure’ being most commonly used. This appears to be consistent with surveys of current and past teaching practice on disability [6, 7, 36] which have found substantial variety in teaching methods. It is clear from this study that there is a variation in teaching content, possibly due to lack of a standardised medical curriculum on this subject [7, 37].

Ten of the sixteen studies were reported as elective. This leads to two concerns; first it increases the risk of bias; as those students taking part in the intervention are likely to be those with a special interest; and therefore, may report more positive attitudes or higher satisfaction. Second, and more importantly, is that this potentially provides a message of ‘unimportance’ of intellectual disability. There is a strong risk that the message to students will be that intellectual disabilities are less important than other areas of medicine where study is mandatory. Students also tend to focus their attention to content and curricula that is assessed, and therefore having this content in an elective format is likely to reduce student engagement and interest [7, 38]. This reinforces a status quo and shows the hidden curriculum [39], whereby some subjects are seen as more or less important as others, despite what might be articulated publicly. This means a considerable number of students are likely to miss out on learning about intellectual disabilities, along with the perception that the needs of those with intellectual disabilities are in some way less important. This has a significant impact on the ability of the future workforce to be able to address health needs of those with intellectual disabilities [7].

An important observation is that many of the studies included lived experience in their teaching interventions. This is consistent with other reviews of intellectual disability medical teaching [7, 40] but was not discussed in older reviews of the same teaching such as that by Kahtan in 1994 [36], suggesting that the involvement of lived experience in teaching is a newer practice. Eleven studies had direct lived experience, with one further study involving parents of someone with an intellectual disability, and two using videos with lived experience. An increase in involvement of lived experience was also found by Trollor and colleagues [7] who carried out a survey of teaching in Australian medical schools. They suggested that increased awareness of the rights of people with a disability provides an opportunity for educational institutions to work more towards the inclusion of people with disabilities in education, and influence greater inclusive teaching practices [7, 40]. Other studies of student views on lived experience involvement [41, 42] have also reported that involving lived experience more widely in medical education has a positive impact on student knowledge and skill, as the encounter is more realistic [41] and influences personal learning [42]. A lived experience component within 14 of the 16 studies appears to be a positive development in the delivery of intellectual disabilities teaching to medical students.

This review showed very little in terms of intervention effectiveness. It did show that classroom-based interventions appeared effective in changing knowledge, but not attitudes. Studies showing significant attitude change were those including face to face contact with someone with an intellectual or developmental disability. However, there were studies with an element of lived experience; such as a placement that showed no change in attitude. This might be due to the nature of the encounter which might be more clinically focussed, and not allow for deeper engagement and communication. Other studies with different intervention types such as placements and panel discussions, were also effective in changing knowledge. Traditionally, lectures may have been favoured as they help to provide factual information [43] and often fit with the status quo, but their use has been challenged in terms of influence on other outcomes, for example the development of positive attitudes [44]. Other delivery methods such as workshops or placements are reportedly more likely to enhance students’ skills in terms of making decisions, as well as modifying attitudes [43, 44].

This scoping review was unable to identify the most effective methods of teaching for a number of reasons. Drawing conclusions regarding effectiveness has been difficult due to low quality assessment of studies, as well as the variety of outcomes measures. For example, several studies measured attitude, but others measured change in skill or practice. It was therefore difficult to identify if a type of education intervention was more effective than another if they did not measure the same outcome. Additionally, many studies indicated positive trends but did not measure statistical significance and have not therefore been assessed as being effective. Challenging methods of evaluation, which were reflected in overall low BEME scores, contributed to a lack of evidence to suggest effectiveness of interventions.

There was also a very low number of studies that were assessed as being higher than a 3 on the Kirkpatrick quality assessment. This is due to the small number of studies that directly assessed learner outcomes past knowledge and attitudes. Low Kirkpatrick scores were also found by Adirim et al. 2021 [6], who looked at current educational interventions on intellectual disability for the postgraduate workforce.

### Limitations

Whilst this study had strengths, several limitations can be listed. First, this study is not a systematic review and therefore there is a lack of standardisation and variability in its conduct [45], however this review did follow systematic principles. The review also only captured studies written in English, or that had evaluated effectiveness, meaning there may be other interventions which are being delivered which we have not included. It is also possible that due to our inclusion criteria some teaching may not have been captured. Despite this, our scoping review addresses a gap in the current literature and gives an overview of current evidence in this area.

## Conclusion

This scoping review has shown a mixed picture of intellectual disability teaching interventions for medical students. With the exception of classroom-based teaching, which is not effective at improving attitudes, it has been difficult to assess effectiveness. There is a need for more consistency in intervention design, and higher quality evaluation of teaching in this area. Our review has however helped draw attention to the broader picture and variation within teaching on this topic area which seems to lack mandated teaching, and further research should focus on updating this review as curriculum changes are implemented over time. A number of countries (Australia, England) have developed capability frameworks for the healthcare workforce along with linked education programme. There is considerable scope for medical schools, and other healthcare training programmes to follow these frameworks in order to ensure that their teaching on the topic is up to date, being given time and importance, and not relying on traditional classroom-based teaching.

## Data Availability

The datasets used and/or analysed during the current study are available from the corresponding author on reasonable request.
